# Associations of Birth Order with Early Adolescent Growth, Pubertal Onset, Blood Pressure and Size: Evidence from Hong Kong’s “Children of 1997” Birth Cohort

**DOI:** 10.1371/journal.pone.0153787

**Published:** 2016-04-18

**Authors:** Man Ki Kwok, Gabriel M. Leung, C. Mary Schooling

**Affiliations:** 1 School of Public Health, Li Ka Shing Faculty of Medicine, The University of Hong Kong, Hong Kong Special Administrative Region, China; 2 City University of New York School of Public Health and Hunter College, New York, NY, United States of America; John Hopkins University School of Medicine, UNITED STATES

## Abstract

**Background:**

Birth order has been proposed as a cardiovascular risk factor, because the lower birth weight and greater infant weight gain typical of firstborns could programme metabolism detrimentally.

**Methods:**

We examined the associations of birth order (firstborn or laterborn) with birth weight-for-gestational age, length/height and body mass index (BMI) z-scores during infancy, childhood, and puberty using generalized estimating equations, with age at pubertal onset using interval-censored regression and with age-, sex- and height-standardized blood pressure, height and BMI z-scores at 13 years using linear regression in a population-representative Chinese birth cohort: “Children of 1997” (n = 8,327).

**Results:**

Compared with laterborns, firstborns had lower birth weight-for-gestational age (mean difference = -0.18 z-score, 95% confidence interval (CI) -0.23, -0.14), lower infant BMI (-0.09 z-score, 95% CI -0.14, -0.04), greater childhood height (0.10 z-score, 95% CI 0.05, 0.14) and BMI (0.08 z-score, 95% CI 0.03, 0.14), but not greater pubertal BMI (0.05 z-score, 95% CI -0.02, 0.11), adjusted for sex, parental age, birthplace, education and income. Firstborns had earlier onset of pubic hair (time ratio = 0.988, 95% CI 0.980, 0.996), but not breast or genitalia, development. Firstborns had greater BMI (0.07 z-score, 95% CI 0.002, 0.15), but not height (0.05 z-score, 95% CI -0.01, 0.11), at 13 years, but similar blood pressure.

**Conclusions:**

Differences by birth order continue into early adolescence with firstborns being heavier with earlier pubic hair development, which could indicate long-term cardiovascular risk.

## Introduction

Differences between groups in cardiovascular disease (CVD) rates at the same level of well-established adult risk factors suggest etiologic gaps, which could be exploited to develop more focused and effective interventions.[1] The World Health Organization (WHO) has endorsed birth weight as an additional CVD risk factor.[2] Nevertheless, the fetal origins hypothesis remains controversial because experimental evidence in humans is limited and the observational evidence appears to be contextually specific, for example less evident in developing populations.[3]

In this situation, examining the effects of other naturally occurring drivers of birth weight and subsequent growth, such as birth order, may help elucidate the role of birth weight and infant growth. Firstborns are different from laterborns in having lower birth weight,[4–6] possibly related to physiological changes in maternal uterine arteries during the first pregnancy facilitating nutrient flow to laterborns,[7] and faster infant growth.[8, 9] They also tend to be taller and/or heavier in early childhood in many settings, including Brazil,[10] the Philippines,[11] Taiwan,[12] New Zealand[13] and Poland,[14] although null associations have been observed in Japan,[15] Denmark[16] and the UK.[17] Given different factors drive growth at different stages (fetal, infant, childhood and puberty),[18] different growth patterns between firstborns and laterborns might be expected to have different long-term consequences.[19] Firstborns tend to have higher blood pressure in childhood and early adolescence as observed in Australia[20] and in older, but not younger, cohorts from Brazil[10, 17] and the UK.[21] They have also been observed to have earlier pubertal onset in a Brazilian cohort.[10] These differences and all subsequent health effects may be the consequences of fetal and infant growth programming metabolism for life,[22, 23] or of childhood growth which may play a larger role in CVD risk factor.[24, 25] Alternatively, these differences in growth could also be a reflection of contextually-specific parenting attitudes, rearing practices, resource sharing and family roles. A decision to have more than one child may be associated with parents’ age, education, income and birthplace. These parental characteristics and the related behaviors may affect the child’s diet, lifestyle, medical care and environmental exposures, thereby generating the observed differences, although a common set of exposures that results in lower birth weight but faster subsequent growth requires somewhat complex explanation. Given worldwide declining trends in fertility, an increasing proportion of the global population are firstborns accentuating the need for understanding its public health implications. Specifically, firstborns represent a particular growth pattern of lower birth weight with faster infant growth. Differences in growth and CVD risk factors between firstborns and laterborns would suggest birth weight and/or infant growth as potentially causal factors of CVD and inform optimal growth patterns for the prevention of CVD. Here, we hypothesized that firstborns are smaller at birth followed by faster subsequent growth and earlier pubertal development, and attain greater current size and higher blood pressure, which might predispose firstborns to higher CVD risks than laterborns.

Most previous studies of parity and CVD risk mainly examined size at a single time point and only one study examined pubertal development. Collating snapshots of growth at different ages from various settings and birth years may not fully reveal growth and development across life-course by birth order, particularly in the presence of sociocultural influences. Hong Kong provides a contrasting, economically developed, non-Western setting to clarify how firstborns differ from laterborns. In Hong Kong, families usually have one to two children with firstborns tending to have younger more educated parents,[26] whereas in more commonly studied Western countries, firstborns have younger but similarly educated parents as laterborns.[27] Intriguingly, children with migrant mothers, mainly from the neighbouring ethnically homogeneous Southern Chinese province, have higher birthweight than those with Hong Kong born mothers,[28] perhaps due to parental self-selection, the healthy migrant effect[29] or gestational diabetes,[30] whereas children with migrant mothers of different ethnicity tend to have lower birthweight than those with native born Caucasian mothers in Western countries.[31] We examined the associations of birth order with length/height and body mass index (BMI) growth at different growth phases, with age at pubertal onset and with blood pressure, height and BMI in early adolescence in a large, population representative Chinese birth cohort, “Children of 1997”.

## Materials and Methods

### Data Source

Hong Kong’s “Children of 1997” birth cohort is a population representative Chinese birth cohort (*n* = 8,327) that covered 88.0% of all births in Hong Kong from April 1, 1997 to May 31, 1997, described in detail elsewhere.[32] The study was initially established to investigate the effect of second-hand smoke exposure on infant health. Families were recruited at the first postnatal visit to any of the 49 Maternal and Child Health Centers in Hong Kong, which parents of all newborns are strongly encouraged to attend for free vaccinations and well-baby checks. Characteristics obtained using a self-administered questionnaire in Chinese at recruitment and subsequent routine visits include maternal and birth characteristics and socioeconomic position. Passive follow-up via record linkage was instituted in 2005 to obtain weight and height from birth to 5 years from the Maternal and Child Health Centers (*n* = 7,999, 96% successful matching); and annual weight and height and bi-annual pubertal status (grade 1 (age 6–7 years) onwards) and blood pressure (grade 5 (age 10–11 years) onwards) from the Student Health Service, Department of Health, which provides free annual check-ups for all school students (*n* = 7,809, 94% successful matching). At the Student Health Service, height without shoes was measured by stadiometer to the nearest 0.1 centimetre and weight without shoes and outer clothing was measured by digital scale to the nearest 0.1 kilogram. BMI was calculated as weight in kilograms divided by height in meters squared. Blood pressure was measured by nurses on the right arm in a seated position after more than 10 minutes of rest with an age and size appropriate cuff size using an automated oscillometric device. Initial systolic or diastolic blood pressure more than the 90^th^ percentile for sex, age and height was double checked by physicians with a sphygmomanometer after 15 minutes of rest and this second measurement was recorded. Pubertal status was visually assessed by physicians for breast (girls) or genitalia (boys) and pubic hair (both sexes) development according to the criteria of Marshall and Tanner.

### Exposure

Information on birth order was obtained from self-administered questionnaires completed mainly by mothers at recruitment (generally shortly after birth) at the Maternal and Child Health Centers. Birth order was categorized as “firstborns” or “laterborns”, because relatively few families had more than two children (11.0%).

### Outcomes

The outcomes were birth weight-for-gestational age z-score, length/height and BMI *z-*scores (standard deviation scores) at different growth phases, age at pubertal onset, and blood pressure, height and BMI z-scores in early adolescence as previously defined.[33–35] Since we do not have birth length, we used birth weight-for-gestational age z-score relative to sex- and gestational age-specific contemporary Hong Kong Chinese infants to proxy fetal growth.[36] Gestational age was calculated from the actual and expected dates of delivery, reported by the mothers or primary caregivers at baseline. The reported expected date of delivery could be based on the date of the last menstrual period and any dating scans. We used length/height or BMI z-scores during 3 to 9 months for infancy (birth-<2 years), 3 to 7 years for childhood (2-<8 years) and 8 to 13 years for puberty (8-<14 years).[37] Each cohort member had up to 11 length/height or BMI measurements taken at about 3 and 9 months for infancy, at 3, 6 and 7 years for childhood, and at 8, 9, 10, 11, 12 and 13 years for puberty. All length/height and BMI measurements were considered as age- and sex-standardized z-scores relative to the 2005 WHO growth standards for 0–5 years[38] and the 2007 WHO growth references for 5–19 years.[39] Age at pubertal onset was defined, as the earliest age when Tanner stage II for breast (girls) or genitalia (boys) and that for pubic hair (both) was recorded. Children with infeasible sequences of pubertal stages, such as pubertal stage II before pubertal stage I (25 girls and 34 boys) were excluded. Systolic and diastolic blood pressure at ~13 years (12-<15 years) z-scores was calculated relative to age-, sex- and height-standardized blood pressure standards from the United States National High Blood Pressure Education Group in 2004.[40] Finally, we considered height and BMI z-score at ~13 years (12-<15 years).

### Statistical analysis

We used multivariable linear regression to assess the adjusted associations of birth order with birth weight-for-gestational age z-score, blood pressure, height and BMI z-scores at ~13 years and multivariable generalized estimating equations with an exchangeable working correlation structure to assess the adjusted associations with height or BMI z-scores during infancy, childhood and puberty, from which mean differences in birth weight-for-gestational age, blood pressure, length/height or BMI z-scores with 95% confidence intervals (CI) are presented. We used multivariable interval-censored regression,[41] with a log-normal distribution, to examine the adjusted associations of birth order with age at pubertal onset, from which time ratios with 95% CI are presented. A time ratio gives the comparison of ages at pubertal onset between groups, so a time ratio greater than 1 indicates older age at pubertal onset, while a time ratio less than 1 indicates younger age at pubertal onset. We also plotted smoothed length/height and BMI from birth to 14 years by birth order using the LOWESS program.

Confounders included were sex, mother’s and father’s age at birth, mother’s and father’s birthplace, highest parental education and household income per head, as categorized in Table 1. We used multiple imputation for missing exposures (among 8,260 cohort members studied, birth order imputed for 3.6%) and confounders (mother’s age for 2.4%, father’s age for 2.5%, mother’s birthplace for 5.4%, father’s birthplace for 4.6%, highest parental education for 2.3% and household income per head for 12.5%) based on a flexible additive regression model with predictive mean matching[42] incorporating data on sex, birth order, mother’s and father’s age, parental height, mother’s and father’s birthplace, highest parental education, household income per head, birth weight-for-gestational age z-score, height, BMI and blood pressure z-scores and maximum age at Tanner stage I and minimum age at Tanner stage II.[43] We summarized the results from 20 imputed datasets into single estimated beta-coefficients or time ratios with CIs adjusted for missing data uncertainty.[44] We also performed an available case analysis, i.e., deleting cases with missing data on variables on an analysis-by-analysis basis, for comparison. Statistical analyses were performed using Stata version 10 (Stata Corp, College station, Texas, USA) and R version 3.0.1 (R Development Core Team, Vienna, Austria).

**Table 1:** Baseline characteristics by birth order for 8,260 adolescents from Hong Kong’s “Children of 1997” birth cohort, Hong Kong, China, 1997–2010.

			Birth order		
			Firstborns	Laterborns	
			(*n* = 3,899)	(*n* = 4,361)	
Characteristics	No.	%	%	%	P-value
Child’s sex					0.06
Female	3913	50.7	51.6	49.9	
Male	4347	56.3	54.8	57.6	
Mother’s age at birth (years)					<0.001
≤24	1036	13.4	21.2	6.4	
25–29	2574	33.3	40.0	27.3	
30–34	3148	40.8	34.9	46.0	
≥35	1503	19.5	10.3	27.8	
Father’s age at birth (years)					<0.001
≤24	317	4.1	7.0	1.5	
25–29	1135	14.7	21.1	8.9	
30–34	2770	35.9	40.8	31.4	
≥35	4038	52.3	37.5	65.7	
Mother’s birthplace					<0.001
Mainland China or elsewhere	3243	42.0	35.4	48.0	
Hong Kong	5017	65.0	71.0	59.6	
Father’s birthplace					<0.001
Mainland China or elsewhere	2538	32.9	24.5	40.4	
Hong Kong	5722	74.1	81.8	67.1	
Parent’s education at recruitment					<0.001
Grade 9 or below	2537	32.9	25.0	40.0	
Grade 10–11	3518	45.6	47.9	43.4	
Grade 12 or above	2206	28.6	33.5	24.1	
Household income per head at					<0.001
recruitment ^a^					
1^st^ quintile	1676	21.7	14.4	28.3	
2^nd^ quintile	1733	22.4	18.4	26.1	
3^rd^ quintile	1633	21.1	19.2	22.9	
4^th^ quintile	1591	20.6	25.9	15.9	
5^th^ quintile	1627	21.1	28.4	14.4	

^a^ Mean (standard deviation) for household income per head at recruitment in quintiles (in Hong Kong dollar; pegged at a rate of 7.8 dollar = 1 U.S. dollar) were 1^st^ quintile: $1,740 (416), 2^nd^ quintile: $2,848 (327), 3^rd^ quintile: $4,365 (557), 4^th^ quintile: $6,812 (874) and 5^th^ quintile: $14,994 (16,341).

### Ethics approval

Since our participants are children, informed consent was obtained from the parents, next of kin, caretakers or guardians (informants) on behalf of the participants by completing the questionnaire at enrollment as approved by The University of Hong Kong Medical Faculty Ethics Committee. Ethical approval for further studies was obtained from the University of Hong Kong-Hospital Authority Hong Kong West Cluster Joint Institutional Review Board and/or the Ethics Committee of the Department of Health, Government of the Hong Kong Special Administrative Region as appropriate.

## Results

Of the original 8,327 cohort members, as of December, 2013, 26 had permanently withdrawn. Among the 8,260 adolescents with at least one measurement of birth weight-for-gestational age, length/height, BMI, blood pressure and pubertal onset, 47.5% were firstborns. Table 1 shows that parents of firstborns were younger, more educated and more likely to be born in Hong Kong and had higher household income per head at recruitment than those of laterborns.

**Fig 1:**
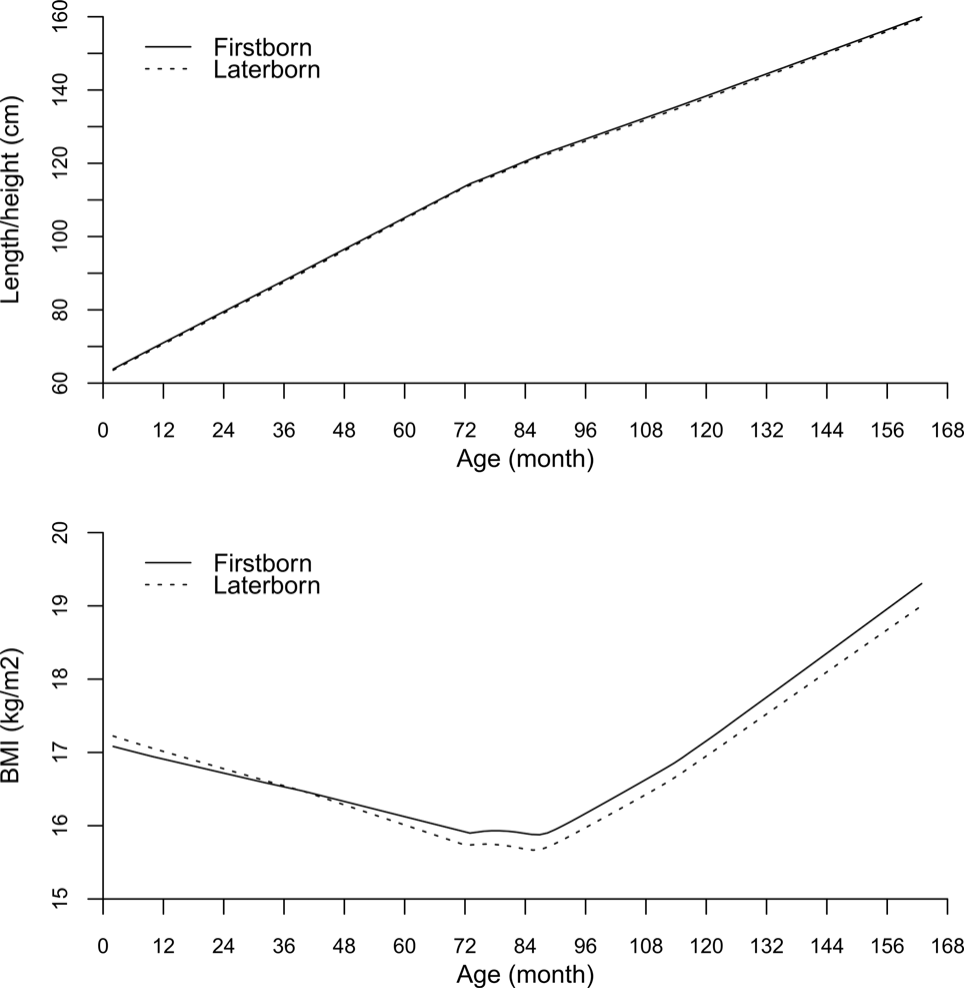
Length/height and body mass index (BMI) growth curves from birth to 14 years by birth order in the Hong Kong’s “Children of 1997” birth cohort, Hong Kong, China, 1997–2010.

Table 2 shows that compared with laterborns, firstborns had lower birth weight-for-gestational age, after adjusting for sex, parental age, birthplace, education and household income. During infancy, firstborns had lower BMI z-score. During childhood, firstborns had greater height and BMI z-scores. During puberty, firstborns had greater height, but not BMI, z-score. Compared with laterborns, firstborns had earlier age at onset of pubic hair development, but not age at onset of breast or genitalia development, after adjusting for sex, parental age, birthplace, education and household income. At ~13 years, firstborns had greater BMI, but not height, z-score, and similar systolic or diastolic blood pressure z-score. The available case analysis produced similar patterns, except the association of birth order with BMI z-scores at 13 years was not statistically significant (S1 Table). Fig 1 shows compared with laterborns, firstborns had similar length/height from early infancy, but lower BMI until about ~3 years after which they had higher BMI than laterborns.

**Table 2:** Adjusted^a^ association of birth order with birth weight-for-gestational age z-score for growth during fetal phase, height and body mass index (BMI) z-scores during infancy, childhood and pubertal phases, age at onset of breast or genitalia or pubic hair development (Tanner stage II) and blood pressure, height and BMI z-scores at 13 years in the Hong Kong’s “Children of 1997” birth cohort, Hong Kong, China, 1997–2010.

Age	Outcomes	Birth order	n	Mean difference^b^	95% CI
Fetal	Birth weight-for-	Firstborns	3,878	-0.18	-0.23, -0.14
	gestational age z-score	Laterborns	4,361	Reference	
Infancy	Length z-score	Firstborns	3,136	0.002	-0.05, 0.05
		Laterborns	3,374	Reference	
	BMI z-score	Firstborns	3,136	-0.09	-0.14, -0.04
		Laterborns	3,374	Reference	
Childhood	Height z-score	Firstborns	3,544	0.10	0.05, 0.14
		Laterborns	3,868	Reference	
	BMI z-score	Firstborns	3,544	0.08	0.03, 0.14
		Laterborns	3,868	Reference	
Puberty	Height z-score	Firstborns	3,592	0.09	0.05, 0.14
		Laterborns	3,994	Reference	
	BMI z-score	Firstborns	3,592	0.05	-0.02, 0.11
		Laterborns	3,994	Reference	
			n	Time ratio	95% CI
	Age at onset of breast or	Firstborns	3,558	0.995	0.987, 1.002
	genitalia development	Laterborns	3,965	1.000	
	Age at onset of pubic	Firstborns	3,577	0.988	0.980, 0.996
	hair development	Laterborns	3,980	1.000	
			n	Mean difference^b^	95% CI
13 years	Systolic blood pressure	Firstborns	2,827	0.02	-0.04, 0.08
	z-score	Laterborns	2,726	Reference	
	Diastolic blood pressure	Firstborns	2,827	-0.01	-0.04, 0.03
	z-score	Laterborns	2,726	Reference	
	Height z-score	Firstborns	2,989	0.05	-0.01, 0.11
		Laterborns	2,979	Reference	
	BMI z-score	Firstborns	2,989	0.07	0.002, 0.15
		Laterborns	2,979	Reference	

^a^ Adjusted for sex, parental age at birth, parental birthplace, highest parental education and household income per head at recruitment

^b^ Mean difference in z-score: 1 unit change in birth weight-for-gestational age z-score is approximated to 370 grams; 1 unit change in height z-score is approximated to 2.3 cm at 9 months, 5.6 cm at 7 years and 7.4 cm at 13 years; 1 unit change in body mass index z-score is approximated to 1.5 kg/m^2^ at 9 months, 1.9 kg/m^2^ at 7 years and 2.7 kg/m^2^ at 13 years; 1 unit change in systolic blood pressure z-score is approximated to 10.6 mmHg and 1 unit change in diastolic blood pressure z-score is approximated to 11.3 mmHg.

## Discussion

In this economically-developed non-Western setting, firstborns had lower birth weight-for-gestational age, lower infant BMI, greater childhood height and BMI, but not greater BMI during puberty than laterborns. By 13 years, firstborns had higher BMI than, but similar height as, laterborns. As a result firstborns had a different trajectory of BMI and height that laterborns with lower BMI in infancy but higher BMI into puberty, they also had somewhat greater childhood and pubertal height which was transient. Firstborns also had earlier age at onset of pubic hair, but not breast or genitalia, development than laterborns. At ~13 years firstborns had similar age-, sex- and height-standardized blood pressure to laterborns.

Several limitations of this study exist. First, we do not have birth length or head circumference as a proxy of fetal growth. Second, pubertal stage was assessed by different physicians. However, a standard guideline for staging and an orchiometer were available and, with our large sample, statistical power is unlikely to have been compromised. Third, using a single blood pressure measurement may slightly overestimate average blood pressure,[45] but systematic overestimation is unlikely to bias the associations of growth with blood pressure. Fourth, we are limited by the latest available measures of growth and blood pressure. However, we considered growth in biologically distinct phases and assessed the role of growth in three of the four growth phases. Fifth, follow-up of our cohort was high, but not complete, so selection bias is possible, although inclusion of adolescents with particular combinations of birth weight, postnatal growth, pubertal development and blood pressure was unlikely. Sixth, unmeasured confounding such as by maternal or fetal outcomes of pregnancy, by family pressure or by infant temperament might affect the child’s growth and parental decisions about having a second child. However, to what extent these unmeasured factors explain the observed differences are unknown. Finally, we lack information on total number of children by the same mother and cannot adjust for family size, although Hong Kong families usually have one to two children.

The finding that firstborns have distinctive growth patterns at different phases across the early life course from laterborns is broadly consistent with previous studies focusing on size at particular ages. Firstborns were lighter at birth adjusted for gestational age.[4–6] They had lower infant BMI, but similar length, unlike infants from rural Gambia[8] or the UK[9] with greater weight. Firstborns had greater childhood height and BMI as seen in Brazil[10] and Philippines,[11] although the greater BMI is not always seen, i.e., in New Zealand,[13] Japan,[15] Denmark,[16] and in younger cohorts of Brazil and the UK.[17] Our findings could indicate that firstborns had greater BMI, but not height, in early adolescence because firstborns start off smaller, any compensatory growth is mainly for BMI occurring in childhood and persists into adolescence.

Our findings of earlier onset of pubic hair, but not breast or genitalia, development among firstborns are similar to the only previous study (from Brazil) that examined pubertal onset,[10] although the two signs of pubertal development were not distinguished. The association of fast infant weight growth with earlier puberty was mediated by childhood height in this birth cohort.[46] Given growth hormone has been associated with earlier puberty,[47] up-regulation of the somatotropic axis controlling growth together with androgens, rather than estradiol,[48] might underlie our observation of greater childhood height and BMI growth together with earlier onset of pubic hair among firstborns. Firstborns in Western settings have earlier age at menarche,[49, 50] consistent with the higher breast cancer incidence among firstborns.[51]

The lack of association of birth order with age-, sex- and height-standardized blood pressure in early adolescence are less consistent with a positive association in Brazilian adolescents[10] and British children[21] or a J-shaped association in Australian children,[20] where blood pressure was not adjusted for height, but are more consistent with no association in Swedish conscripts[52] and Brazilian adults.[53] Our findings suggest birth weight and infant growth play little role in early adolescent blood pressure, inconsistent with fetal and infant metabolic programming,[22, 23] but more consistent with other growth phases playing a larger role.[24] Life history theory posits that resources are partitioned between growth, maturation and maintenance to maximize reproductive success,[54] with any metabolic effects as a by-product; no particular reason suggests firstborns pursue a different strategy from laterborns.

Alternatively, parents may pay more attention to firstborns and feed or raise them differently from laterborns. Many firstborns may be only children, whilst laterborns are not. A difference in intelligent quotient between first-borns and laterborns appears to be socially rather than biologically driven, because second-borns whose older sibling dies in infancy have similar intelligent quotient as firstborns.[55] In this cohort, mothers of firstborns were more likely to initiate breastfeeding. Differences in early growth patterns between firstborns and laterborns might reflect family resource allocation Firstborns naturally have their parents’ undivided attention at least in the first year and will obtain more resources. With the birth of other siblings, it is theorized that parents may still preferentially allocate resources to firstborns over laterborns in favourable conditions, perhaps due to earlier reproductive maturity.[24] In Hong Kong, preferences for longer intervals between births[25] could have allowed longer period of undivided attention for firstborns. Moreover, domestic helpers and grandparents are the main caregivers, particularly among higher income families in Hong Kong;[26] such informal child care (compared with parental care) was positively associated with childhood adiposity.[27] Firstborns might tend to have greater adiposity in our setting in view of the socio-cultural childrearing practice, although this unlikely explain our findings of lower birth weight and lower infant BMI among firstborns. Alternatively, migrant mothers may exert different childrearing practice from Hong Kong born mothers, as shown by the opposite socioeconomic patterning of childhood adiposity.[56] However, in this study, laterborns were more common in families with migrant mothers and thus would be expected to have greater adiposity,[56] which was opposite to our observed association. Moreover, we adjusted for parents’ age, birthplace, education and income, hence our observed greater adiposity and earlier pubic hair development in firstborns are unlikely explained by different rearing attitudes or practice by migrant status. Nonetheless, such non-salient differences could be open to unmeasured or residual socioeconomic confounding. However, our specific associations of birth order might suggest some degree of biological plausibility and distinguish a causal hypothesis from a non-causal hypothesis because residual confounding would be expected to generate systematic associations.

## Conclusions

Firstborns were heavier into early adolescence. They also had earlier pubic hair, but not breast or genitalia, development, perhaps due to biological factors that underlie growth and pubic hair development and are more common in firstborns. Whether these differences extend into adulthood and affect cardiovascular risk remains to be elucidated in a range of settings with different confounding structures. Our study highlights the importance of replicating observations in different settings that allows thorough investigation of the underlying biological mechanism to presage any consequences in populations with increasing proportion of firstborns. Equally important, our findings showed very modest differences in birth weight (-0.18 z-score, equivalent to ~67 grams) or BMI z-scores between firstborns and laterborns until adolescence (e.g. for BMI at 13 years, 0.07 z-score, equivalent to ~0.19 kg/m^2^). Based on 1 kilogram higher birth weight is associated with 16% lower risk of ischemic heart disease[57] and 1 kilogram higher adult BMI is associated with 4–5% higher risk of coronary heart disease,[58] our estimates could translate to ~1% lower risk of CVD. Given CVD is the leading cause of morbidity and mortality,[59] these are important at a population level, particularly as firstborns are forming an increasing proportion of the population in developed or rapidly developing settings where family size is falling with improved living conditions. These associations also add etiological insights for more complete understanding of the life course development of CVD, implying that the typical growth pattern of firstborns (lower birth weight and greater subsequent adiposity) may be suboptimal for the prevention of CVD.

## Supporting Information

S1 Table Available case analysis for adjusted association of birth order with birth weight-for-gestational age z-score for growth during fetal phase, length/height and body mass index (BMI) z-scores during infancy, childhood and pubertal phases, age at onset of breast or genitalia or pubic hair development (Tanner stage II) and blood pressure, height and BMI z-scores at 13 years in the Hong Kong’s “Children of 1997” birth cohort, Hong Kong, China, 1997–2010. (DOC)
