# Supplementary material for: Associations of Birth Order with Early Adolescent Growth, Pubertal Onset, Blood Pressure and Size: Evidence from Hong Kong’s “Children of 1997” Birth Cohort
Source: PLoS One. 2016 Apr 18;11(4):e0153787. doi: 10.1371/journal.pone.0153787 (PMC4835083; doi:10.1371/journal.pone.0153787)
Supplement: S1 Table — (DOC) [file pone.0153787.s001.doc]

S1 Table. Available case analysis for adjusted^a^ association of birth order with birth weight-for-gestational age z-score for growth during fetal phase, length/height and body mass index (BMI) z-scores during infancy, childhood and pubertal phases, age at onset of breast or genitalia or pubic hair development (Tanner stage II) and blood pressure, height and BMI z-scores at 13 years in the Hong Kong’s “Children of 1997” birth cohort, Hong Kong, China, 1997-2010

| Age | Outcomes | Birth order | n | Mean difference^b^ | 95% CI |
| --- | --- | --- | --- | --- | --- |
| Fetal | Birth weight-for-gestational age z-score | Firstborns | 3,152 | -0.21 | -0.26, -0.16 |
|  |  | Laterborns | 3,454 | Reference |  |
| Infancy | Length z-score | Firstborns | 2,738 | -0.02 | -0.07, 0.03 |
|  |  | Laterborns | 2,864 | Reference |  |
|  | BMI z-score | Firstborns | 2,738 | -0.12 | -0.17, -0.06 |
|  |  | Laterborns | 2,864 | Reference |  |
| Childhood | Height z-score | Firstborns | 2,970 | 0.06 | 0.01, 0.11 |
|  |  | Laterborns | 3,135 | Reference |  |
|  | BMI z-score | Firstborns | 2,970 | 0.06 | 0.0005, 0.12 |
|  |  | Laterborns | 3,135 | Reference |  |
| Puberty | Height z-score | Firstborns | 3,000 | 0.07 | 0.02, 0.13 |
|  |  | Laterborns | 3,230 | Reference |  |
|  | BMI z-score | Firstborns | 3,000 | 0.02 | -0.05, 0.08 |
|  |  | Laterborns | 3,230 | Reference |  |
|  |  |  |  |  |  |
|  |  |  | n | Time ratio | 95% CI |
|  | Age at onset of breast or genitalia | Firstborns | 2,968 | 0.996 | 0.988, 1.004 |
|  | development | Laterborns | 3,204 | 1.000 |  |
|  | Age at onset of pubic hair development | Firstborns | 2,982 | 0.988 | 0.980, 0.997 |
|  |  | Laterborns | 3,214 | 1.000 |  |
|  |  |  |  |  |  |
|  |  |  | n | Mean difference^b^ | 95% CI |
| 13 years | Systolic blood pressure z-score | Firstborns | 2,369 | 0.04 | -0.02, 0.11 |
|  |  | Laterborns | 2,238 | Reference |  |
|  | Diastolic blood pressure z-score | Firstborns | 2,369 | 0.003 | -0.03, 0.04 |
|  |  | Laterborns | 2,238 | Reference |  |
|  | Height z-score | Firstborns | 2,491 | 0.04 | -0.02, 0.10 |
|  |  | Laterborns | 2,440 | Reference |  |
|  | BMI z-score | Firstborns | 2,491 | 0.04 | -0.03, 0.12 |
|  |  | Laterborns | 2,440 | Reference |  |

\

^a^ Adjusted for sex, parental age at birth, parental birthplace, highest parental education and household income per head at recruitment

^b^ Mean difference in z-score: 1 unit change in birth weight-for-gestational age z-score is approximated to 370 grams; 1 unit change in height z-score is approximated to 2.3 cm at 9 months, 5.6 cm at 7 years and 7.4 cm at 13 years ; 1 unit change in body mass index z-score is approximated to 1.5 kg/m^2^ at 9 months, 1.9 kg/m^2^ at 7 years and 2.7 kg/m^2^ at 13 years; 1 unit change in systolic blood pressure z-score is approximated to 10.6 mmHg and 1 unit change in diastolic blood pressure z-score is approximated to 11.3 mmHg.
